# Health-related quality of life in premenopausal women with hormone-receptor-positive, HER2-negative advanced breast cancer treated with ribociclib plus endocrine therapy: results from a phase III randomized clinical trial (MONALEESA-7)

**DOI:** 10.1177/1758835920943065

**Published:** 2020-07-26

**Authors:** Nadia Harbeck, Fabio Franke, Rafael Villanueva-Vazquez, Yen-Shen Lu, Debu Tripathy, Louis Chow, Govind K Babu, Young-Hyuck Im, David Chandiwana, Anil Gaur, Brad Lanoue, Karen Rodriguez-Lorenc, Aditya Bardia

**Affiliations:** Breast Center, Department of Obstetrics and Gynecology and CCCLMU, Ludwig-Maximilians University Munich (LMU), Marchioninistrasse 15, München, 81377, Germany; Hospital de Caridade de Ijuí, CACON, Ijuí, Brazil; Institut Català d’Oncologia, Hospital de Sant Joan Despí Moisès Broggi, Barcelona, Spain; National Taiwan University Hospital, Taipei; The University of Texas MD Anderson Cancer Center, Houston, TX, USA; Organisation for Oncology and Translational Research, Hong Kong; HCG Curie Centre of Oncology and Kidwai Memorial Institute of Oncology, Bangalore, Karnataka, India; Samsung Medical Center, Sungkyunkwan University School of Medicine, Seoul, Republic of Korea; Novartis Pharmaceuticals Corporation, East Hanover, NJ, USA; Novartis Healthcare Pvt Ltd, Hyderabad, India; Novartis Pharmaceuticals Corporation, East Hanover, NJ, USA; Novartis Pharmaceuticals Corporation, East Hanover, NJ, USA; Massachusetts General Hospital Cancer Center, Harvard Medical School, Boston, MA, USA

**Keywords:** breast cancer, endocrine therapy, quality of life, ribociclib

## Abstract

**Background:**

This analysis evaluated patient-reported outcomes (PROs) to assess health-related quality of life (HRQoL) in the phase III MONALEESA-7 trial, which previously demonstrated improvements in progression-free survival (PFS) and overall survival (OS) with ribociclib (cyclin-dependent kinase 4/6 inhibitor) + endocrine therapy (ET) compared with placebo + ET in pre- and perimenopausal patients with hormone-receptor-positive, HER2-negative (HR+/HER2−) advanced breast cancer (ABC).

**Methods:**

The European Organisation for Research and Treatment of Cancer (EORTC) Quality of Life questionnaire C30 (QLQ-C30) and the EQ-5D-5L were used to evaluate HRQoL.

**Results:**

EORTC QLQ-C30 assessments were evaluable for 335 patients in the ribociclib arm and 337 patients in the placebo arm. Adherence rates at baseline and ⩾1 postbaseline time point were 90% and 83%, respectively. Patients treated with ribociclib + ET had a longer time to deterioration (TTD) ⩾ 10% in global HRQoL {hazard ratio (HR), 0.67 [95% confidence interval (CI), 0.52–0.86]}. TTD ⩾ 10% in global HRQoL was delayed in ribociclib-treated patients without *versus* with disease progression [HR, 0.31 (95% CI, 0.21–0.48)]. TTD ⩾ 10% in pain was longer with ribociclib + ET than with placebo + ET [HR, 0.65 (95% CI, 0.45–0.92)]. Patients who received a nonsteroidal aromatase inhibitor experienced similar benefits with ribociclib *versus* placebo in global HRQoL and pain.

**Conclusion:**

HRQoL was maintained longer in patients who received ribociclib + ET *versus* placebo + ET. These data, combined with previously reported improvements in PFS and OS, support a strong clinical benefit-to-risk ratio with ribociclib-based treatment in pre- and perimenopausal patients with HR+/HER2− ABC.

## Introduction

Endocrine therapy with ovarian suppression plus a cyclin-dependent kinase 4/6 (CDK4/6) inhibitor is a recommended first-line treatment for premenopausal women with hormone-receptor-positive (HR+)/human epidermal growth factor receptor 2-negative (HER2−) advanced breast cancer (ABC)^1,2^; however, treatment resistance and disease progression ultimately occur. In addition to these potential complexities of breast cancer in premenopausal women, maintaining quality of life (QoL) in a younger patient population presents a unique treatment challenge. ABC is associated with reduced QoL; however, treatment may improve or at least stabilize global QoL and specific disease-related symptoms.^3^

MONALEESA-7 is a phase III trial of first-line endocrine-based therapy with ribociclib or placebo with goserelin and a nonsteroidal aromatase inhibitor (NSAI; letrozole or anastrozole) or tamoxifen in premenopausal patients with ABC.^4^ This is the only phase III trial to prospectively assess a CDK4/6 inhibitor in combination with endocrine therapy in a population of exclusively premenopausal patients with HR+/HER2− ABC. In MONALEESA-7, progression-free survival (PFS) was prolonged with ribociclib *versus* placebo {median, 23.8 *versus* 13.0 months; hazard ratio (HR), 0.55 [95% confidence interval (CI), 0.44–0.69]; *p* < 0.0001}. The addition of ribociclib to endocrine therapy also resulted in statistically significant longer overall survival (OS), with a 29% reduction in the relative risk of death compared with placebo [HR, 0.71 (95% CI, 0.54–0.95); *p* = 0.00973] and estimated OS rates of 70.2% (95% CI, 63.5–76.0%) *versus* 46.0% (95% CI, 32.0–58.9%) at 42 months.^5^ A consistent OS benefit was observed with ribociclib over placebo in the subgroup of patients who received a nonsteroidal aromatase inhibitor (NSAI) as endocrine therapy partner [HR, 0.70 (95% CI, 0.50–0.98)]. The safety profile was generally manageable with dose modifications and concomitant medications. In the ribociclib and placebo arms, 4% and 3%, respectively, of patients discontinued therapy due to adverse events.^4^

Because QoL is among the most important considerations for patients with cancer, one of the predefined secondary objectives of the MONALEESA-7 study was evaluation of patient-reported outcomes (PROs) for health-related QoL (HRQoL). We report these data, which were analyzed from the recent final data cutoff used to evaluate OS.

## Methods

The details of the MONALEESA-7 trial design and participants have been described previously.^4^ Written informed consent was obtained from all patients at enrollment. The study was approved by each participating site’s institutional review board or independent ethics committee (Supplemental Table S1). The trial was performed in accordance with the Good Clinical Practice guidelines and the Declaration of Helsinki. This study is registered [ClinicalTrials.gov identifier: NCT02278120]. The data cutoff date for this analysis was 30 November 2018.

### Patient-reported outcomes

Patients completed questionnaires in person at the beginning of each visit. PROs were evaluated based on the European Organisation for Research and Treatment of Cancer (EORTC) Quality of Life questionnaire core 30 (QLQ-C30) and Breast cancer module (QLQ-BR23). The EQ-5D-5L was used to evaluate PRO measures of HRQoL, functioning, disease symptoms, and treatment-related side effects.

Time to deterioration (TTD) ⩾ 10% in the global health status/QoL scale and secondary PRO variables of the EORTC QLQ-C30 were analyzed based on the 10-point threshold, which is considered a reference for clinical meaningfulness.^6,7^ Definitive 10% deterioration was defined as a worsening in QoL score by ⩾10% compared with baseline, with no later improvement above this threshold observed during the treatment period, or death due to any cause.

### Statistical analyses

TTD was compared between the two treatment arms using a stratified log-rank test at a 1-sided 2.5% level of significance. Survival distributions were analyzed using the Kaplan–Meier method. A stratified Cox regression was used to estimate the HR for TTD, along with 2-sided 95% CI. Descriptive statistics were used to summarize the scores from study PRO assessments at each scheduled assessment time point. Change from baseline in all subscales was analyzed using a linear mixed-effects model that included the following factors: treatment, stratification factors, and baseline score; this analysis only included assessments up to the time point at which ⩾50 patients were evaluable in each treatment arm.

## Results

### Patient disposition

As previously reported at the data cutoff of 30 November 2018, there were 116 patients (34.6%) continuing treatment in the ribociclib arm and 57 patients (16.9%) continuing treatment in the placebo arm.^5^ At data cutoff, 219 patients (65.4%) in the ribociclib arm and 280 patients (83.1%) in the placebo arm had discontinued treatment. The main cause of treatment discontinuation among these patients was progressive disease (51.6% and 68.2% in the ribociclib and placebo arms, respectively).

### Quality of life

**Figure 1.**
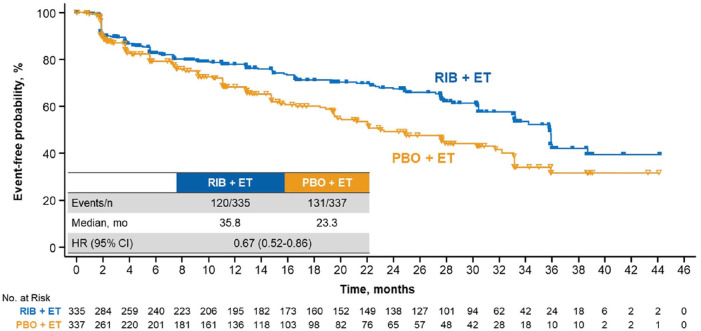
TTD ⩾10% in global HRQoL in patients treated with RIB *versus* PBO. CI, confidence interval; ET, endocrine therapy; HR, hazard ratio; HRQoL, health-related quality of life; mo, months; PBO, placebo; RIB, ribociclib; TTD, time to deterioration.

**Figure 2.**
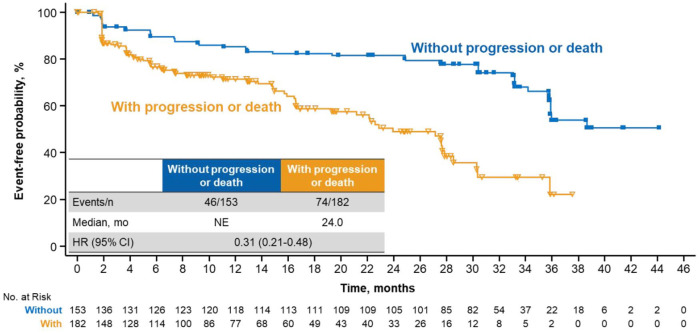
TTD ⩾10% in global HRQoL in patients treated with RIB with *versus* without disease progression. CI, confidence interval; HR, hazard ratio; HRQoL, health-related quality of life; mo, months; NE, not estimable; RIB, ribociclib; TTD, time to deterioration.

QoL was assessed at baseline and throughout treatment; the EORTC QLQ-C30 was completed at baseline and ⩾1 postbaseline time point by 90% and 83% of patients in the ribociclib and placebo arms, respectively. TTD ⩾ 10% in global HRQoL was significantly delayed with ribociclib *versus* placebo [median, 35.8 *versus* 23.3 months, respectively; HR, 0.67 (95% CI, 0.52–0.86)] (Figure 1). Analyses conducted at deterioration cutoffs of 5% and 15% conveyed similar findings: TTD ⩾ 5% [median, 35.8 *versus* 23.3 months; HR, 0.65 (95% CI, 0.51–0.84)] and ⩾15% [median, 38.7 *versus* 33.1 months; HR, 0.69 (95% CI, 0.52–0.92)] were consistently delayed with ribociclib *versus* placebo, respectively (Supplemental Figure S1A and B). Patients treated with ribociclib without disease progression experienced delayed TTD ⩾ 10% in global HRQoL *versus* patients with disease progression [median, not estimable (NE) *versus* 24.0 months; HR, 0.31 (95% CI, 0.21–0.48)] (Figure 2).

Global health status was also assessed as change from baseline in a linear mixed-effects model that accounted for treatment, stratification factors, and baseline health status score (Supplemental Figure S2). The baseline global health status scores of 64.7 in the ribociclib arm and 65.1 in the placebo arm were similar to the 62.9 observed in a large general (i.e., not cancer specific) population of women aged 40–49 years in North America and Europe.^8^ After 25 cycles, which corresponds with the median treatment duration of 2 years in the ribociclib arm,^5^ the mean score changes from baseline in the ribociclib arm (*n* = 164 evaluable) and the placebo arm (*n* = 92 evaluable) were +3.9 and +2.2 points, respectively. At the end of treatment, the mean score changes from baseline in the ribociclib arm (*n* = 182 evaluable) and the placebo arm (*n* = 236 evaluable) were −4.0 and −3.2 points, respectively.

**Figure 3.**
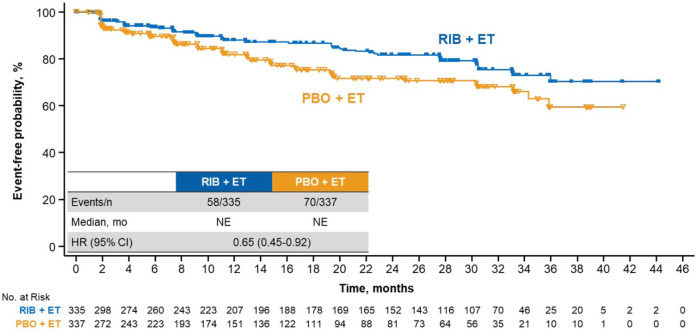
TTD ⩾10% in pain in patients treated with RIB *versus* PBO. CI, confidence interval; ET, endocrine therapy; HR, hazard ratio; mo, months; NE, not estimable; PBO, placebo; RIB, ribociclib; TTD, time to deterioration.

**Figure 4.**
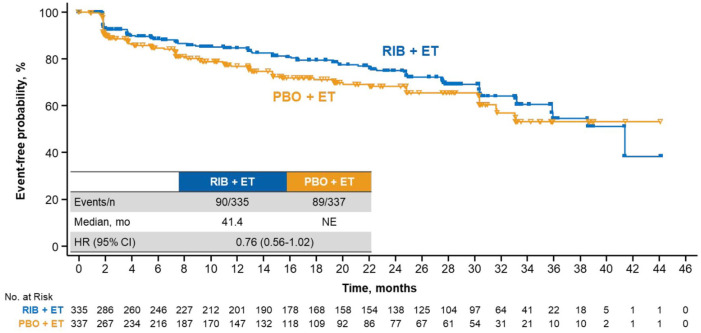
TTD ⩾10% in fatigue in patients treated with RIB *versus* PBO. CI, confidence interval; ET, endocrine therapy; HR, hazard ratio; mo, months; NE, not estimable; PBO, placebo; RIB, ribociclib; TTD, time to deterioration.

Ribociclib treatment led to a longer maintenance than placebo in key subdomains of the EORTC QLQ-C30 questionnaire, including pain and fatigue. The ribociclib arm demonstrated a delay in TTD ⩾ 10% in pain *versus* placebo [median, NE in either arm; HR, 0.65 (95% CI, 0.45–0.92)] (Figure 3). A consistent benefit was observed in TTD ⩾ 10% in fatigue, although the effect size was not as large as that observed for global QoL or pain (TTD ⩾ 10% in fatigue: HR, 0.76 [95% CI, 0.56–1.02]) (Figure 4). Similar to that of the global health status, the trends TTD ⩾ 10% in physical, emotional, and social functioning also favored ribociclib treatment (Supplemental Figure S3).

**Figure 5.**
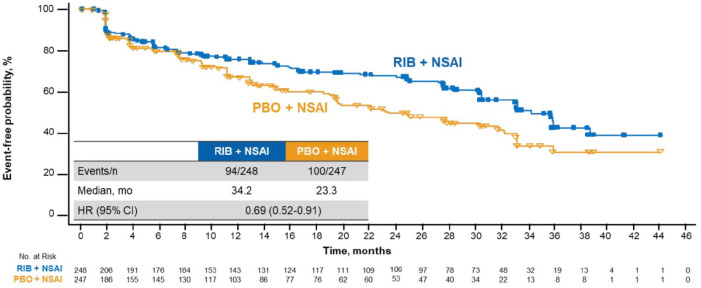
TTD ⩾ 10% in global HRQoL in patients treated with RIB *versus* PBO plus an NSAI. CI, confidence interval; HR, hazard ratio; HRQoL, health-related quality of life; mo, months; NSAI, nonsteroidal aromatase inhibitor; PBO, placebo; RIB, ribociclib; TTD, time to deterioration.

**Figure 6.**
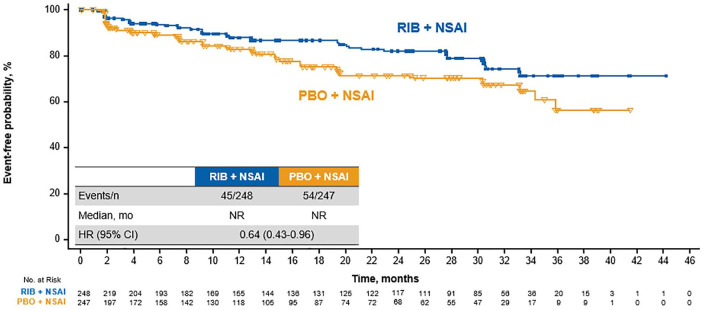
TTD ⩾ 10% in pain in patients treated with RIB *versus* PBO plus an NSAI. CI, confidence interval; HR, hazard ratio; mo, months; NSAI, nonsteroidal aromatase inhibitor; PBO, placebo; RIB, ribociclib; NE, not estimable; TTD, time to deterioration.

Ribociclib is approved for use in combination with an NSAI in premenopausal patients; therefore, analyses were performed on global health status and subdomains of the EORTC QLQ-C30 questionnaire in the MONALEESA-7 NSAI cohort (ribociclib arm, *n* = 248; placebo, *n* = 247). Results in the NSAI cohort were generally similar to those observed in the overall patient population. Median TTD ⩾ 10% in global health status was 34.2 months in the ribociclib arm *versus* 23.3 months in the placebo arm [HR, 0.69 (95% CI, 0.52–0.91)] (Figure 5). In the key subdomain of pain, median TTD ⩾ 10% was not reached in either treatment arm; however, the hazard ratio indicated a benefit with ribociclib [HR, 0.64 (95% CI, 0.43–0.96)] (Figure 6). Fatigue also appeared to be numerically better, albeit to a lesser extent, with ribociclib than with placebo (median TTD ⩾ 10%, 41.4 months *versus* NE; HR 0.78 [95% CI, 0.56–1.10]) (not shown).

## Discussion

The primary efficacy report and a more recent OS report of the MONALEESA-7 trial have demonstrated statistically significant benefits in PFS and OS with ribociclib plus endocrine therapy over placebo plus endocrine therapy, as well as a manageable safety profile,^4,5^ supporting the clinical benefit of the regimen. Another key aspect of clinical benefit is HRQoL, which was assessed *via* PROs as a secondary endpoint in the MONALEESA-7 trial. HRQoL is particularly relevant for premenopausal patients because younger patients may face substantial unease about the impact of breast cancer on their ability to care for family and manage careers, among other factors.^9^ In this analysis, HRQoL was maintained throughout the study treatment, and the TTD in global health status as well as the key subdomains of pain and fatigue were delayed with ribociclib compared with placebo. Similar results were observed with ribociclib over placebo in the cohort of patients who received an NSAI.

In MONALEESA-7, the longer PFS in the ribociclib arm over the placebo arm was accompanied by an improvement in global health status over placebo. That is, patients receiving ribociclib had an improved QoL during treatment as well as a longer time without disease progression, which are likely the key factors driving the longer TTD ⩾ 10% in global health status with ribociclib.

Clinical benefit is generally thought to be based on the three pillars of efficacy, safety, and HRQoL. PFS benefits have been observed with ribociclib combinations in all three MONALEESA trials.^4,10–12^ In the MONALEESA-3 and MONALEESA-7 trials, ribociclib combinations also demonstrated statistically significant benefits in OS^5,13^ (OS results from MONALEESA-2 had not been presented as of this writing because the data were not yet mature). The tolerability profile of ribociclib has been well established, and adverse events are generally manageable. Consistent with the maintenance of QoL with ribociclib in the MONALEESA-7 trial, separate analyses of PROs demonstrated that QoL was also maintained throughout treatment with the addition of ribociclib to letrozole in the MONALEESA-2 trial and the addition of ribociclib to fulvestrant in the MONALEESA-3 trial.^14,15^

In this analysis of MONALEESA-7, HRQoL was maintained for a longer duration with ribociclib than with placebo, with a HR of 0.67 (95% CI, 0.52–0.86) for TTD in EORTC QLQ-C30 global health status. To place these data into context, it should be noted that the HRQoL benefit of adding CDK4/6 inhibitors to endocrine therapy was less pronounced in other phase III trials, although cross-trial comparisons must be made with caution. For example, the HR for abemaciclib over placebo for time to sustained deterioration in EORTC QLQ-C30 global health status [based on a ⩾10% decrease (similar to TTD in this analysis) followed by all subsequent scores meeting the minimally important difference criteria compared with baseline] in the MONARCH-2 trial was 0.80 (95% CI, 0.63–1.02).^16^ Also, although results for TTD in EORTC scores had not been reported for MONARCH-3 as of this writing, an analysis of HRQoL revealed that the addition of abemaciclib to an NSAI did not result in statistically significant and clinically meaningful differences in patient-reported global health status.^17^ Additionally, while QoL results for palbociclib plus letrozole in the PALOMA-2 study did not include EORTC QLQ-C30 global health status, results for TTD in Functional Assessment of Cancer Therapy-Breast questionnaire scores were similar between the palbociclib and placebo arms [HR, 0.88 (95% CI, 0.67–1.16)].^18^ The results for MONARCH-3 and PALOMA-2 are particularly relevant because these trials are also investigating the addition of a CDK4/6 inhibitor to an NSAI in the first-line setting, albeit primarily in postmenopausal patients.

Given the substantial clinical benefit in terms of PFS and OS improvements seen in MONALEESA-7, it is noteworthy that ribociclib in combination with endocrine therapy also improves QoL in this patient population, which may include some women experiencing side effects from ovarian suppression. These data support the overall clinical and HRQoL benefits of CDK4/6 inhibitor-based combination therapy in treating premenopausal and perimenopausal patients with HR+/HER2− ABC.

## Supplemental Material

Harbeck_TAMO,_Supplemental_Material_ML-7_PRO_Post-Review_22June2020 – Supplemental material for Health-related quality of life in premenopausal women with hormone-receptor-positive, HER2-negative advanced breast cancer treated with ribociclib plus endocrine therapy: results from a phase III randomized clinical trial (MONALEESA-7) Click here for additional data file. Supplemental material, Harbeck_TAMO,_Supplemental_Material_ML-7_PRO_Post-Review_22June2020 for Health-related quality of life in premenopausal women with hormone-receptor-positive, HER2-negative advanced breast cancer treated with ribociclib plus endocrine therapy: results from a phase III randomized clinical trial (MONALEESA-7) by Nadia Harbeck, Fabio Franke, Rafael Villanueva-Vazquez, Yen-Shen Lu, Debu Tripathy, Louis Chow, Govind K Babu, Young-Hyuck Im, David Chandiwana, Anil Gaur, Brad Lanoue, Karen Rodriguez-Lorenc and Aditya Bardia in Therapeutic Advances in Medical Oncology
