# Supplementary material for: Health-related quality of life in premenopausal women with hormone-receptor-positive, HER2-negative advanced breast cancer treated with ribociclib plus endocrine therapy: results from a phase III randomized clinical trial (MONALEESA-7)
Source: Ther Adv Med Oncol. 2020 Jul 26;12:1758835920943065. doi: 10.1177/1758835920943065 (PMC7385843; doi:10.1177/1758835920943065)
Supplement: Harbeck_TAMO,_Supplemental_Material_ML-7_PRO_Post-Review_22June2020 – Supplemental material for Health-related quality of life in premenopausal women with hormone-receptor-positive, HER2-negative advanced breast cancer treated with ribociclib plus endocrine therapy: results from a phase III randomiz [file Harbeck_TAMO,_Supplemental_Material_ML-7_PRO_Post-Review_22June2020.pdf]

Data Sharing Statement

Harbeck N, et al. Health-Related Quality of Life in Premenopausal Women With Hormone Receptor-Positive, HER2-negative Advanced Breast Cancer Treated With Ribociclib Plus Endocrine Therapy: Results From a Phase 3 Randomized Clinical Trial (MONALEESA-7).

**Question**	**Authors' Response**
Will individual participant data be available (including data dictionaries)?	No
What data in particular will be shared?	-
What other documents will be available?	-
When will data be available (start and end dates)?	-
With whom?	-
For what types of analyses?	-
By what mechanism will data be made available?	-

Supplemental Figures

Supplemental Figure 1. Time to deterioration ≥ 5% (A) and ≥ 15% (B) in global health-related quality of life. ET, endocrine therapy; HR, hazard ratio; PBO, placebo; RIB, ribociclib.

A.

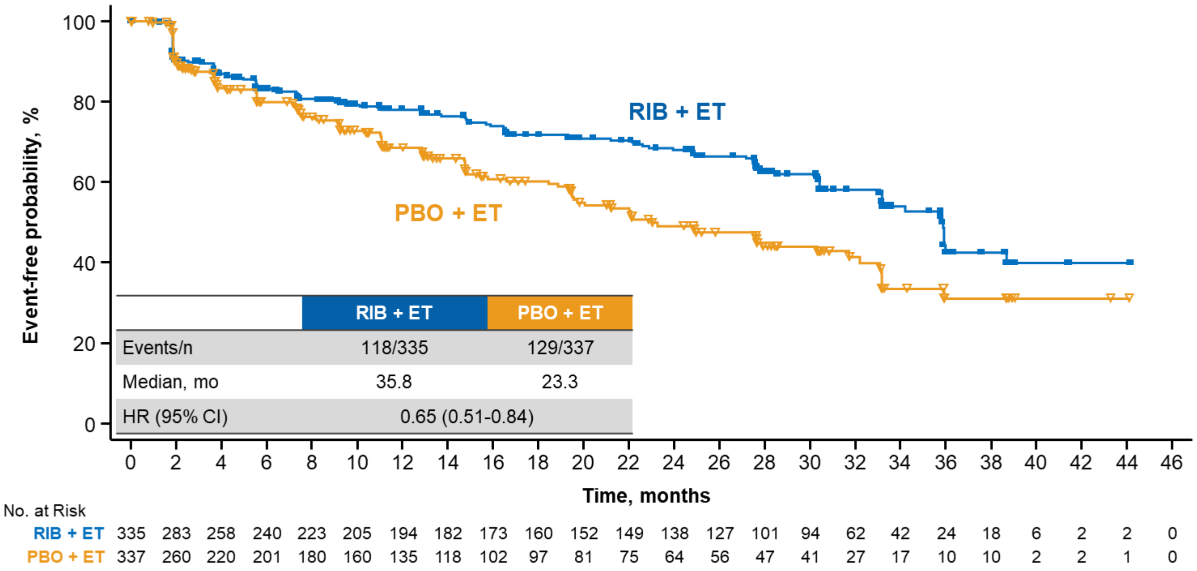

B.

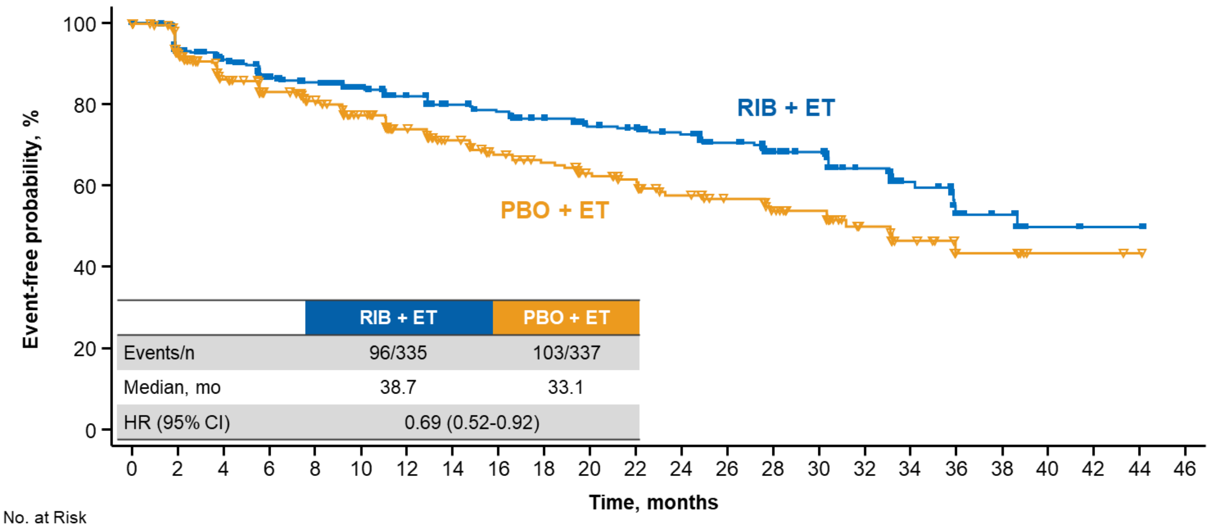

No. at Risk

RIB + ET	335	289	267	247	234	214	200	186	179	167	157	154	144	132	107	100	63	44	25	19	6	2	2	0
PBO + ET	337	267	226	205	185	164	140	123	109	102	87	81	69	62	53	47	29	20	10	10	2	2	1	0

Supplemental Figure 2. Change from baseline in global health-related quality of life. C, cycle; D, day; EOT, end of treatment; ET, endocrine therapy; LS, least squares; PBO, placebo; RIB, ribociclib.

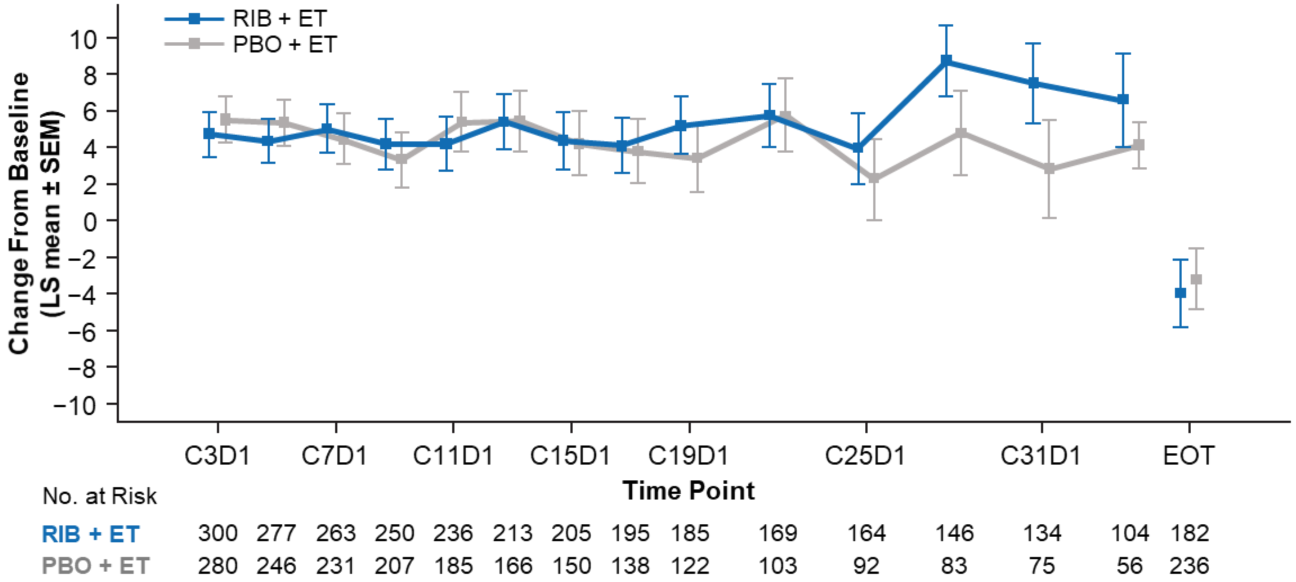

Supplemental Figure 3. Time to deterioration ≥ 10% in physical functioning (A), emotional functioning (B), and social functioning (C). ET, endocrine therapy; HR, hazard ratio; PBO, placebo; RIB, ribociclib.

A.

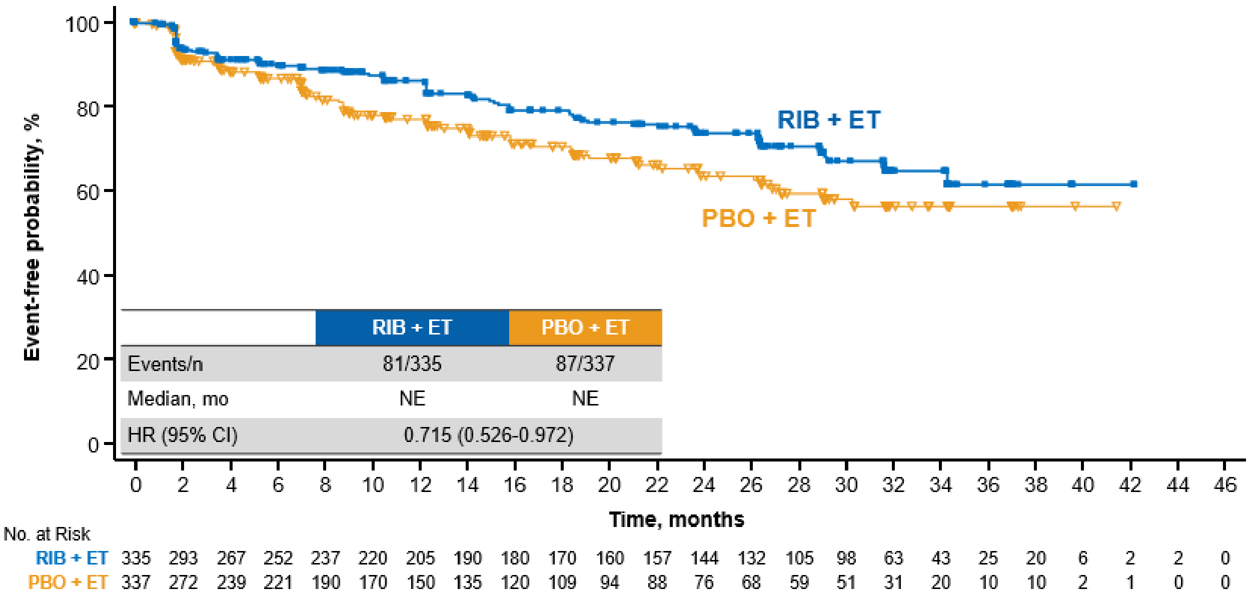

B.

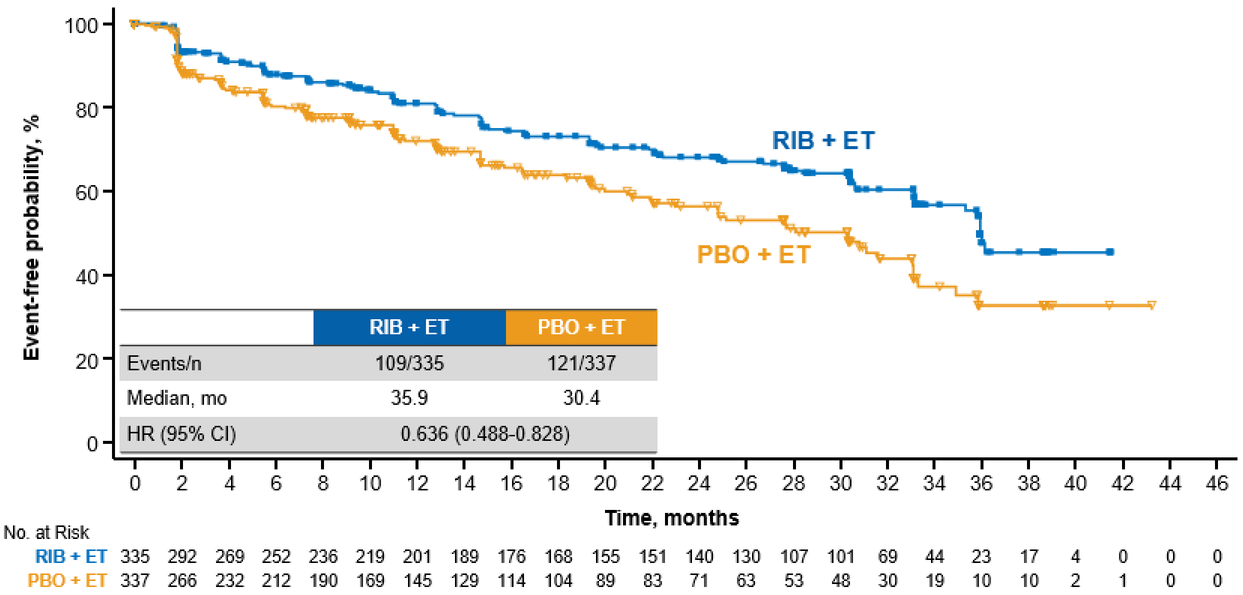
100
80
60
40
20
0
Event-free probability, %
RIB + ET
PBO + ET
RIB + ET | PBO + ET
Events/n | 109/335 | 121/337
Median, mo | 35.9 | 30.4
HR (95% CI) | 0.636 (0.488-0.828)
0 2 4 6 8 10 12 14 16 18 20 22 24 26 28 30 32 34 36 38 40 42 44 46
Time, months
No. at Risk
RIB + ET 335 292 269 252 236 219 201 189 176 168 155 151 140 130 107 101 69 44 23 17 4 0 0 0
PBO + ET 337 266 232 212 190 169 145 129 114 104 89 83 71 63 53 48 30 19 10 10 2 1 0 0

C.

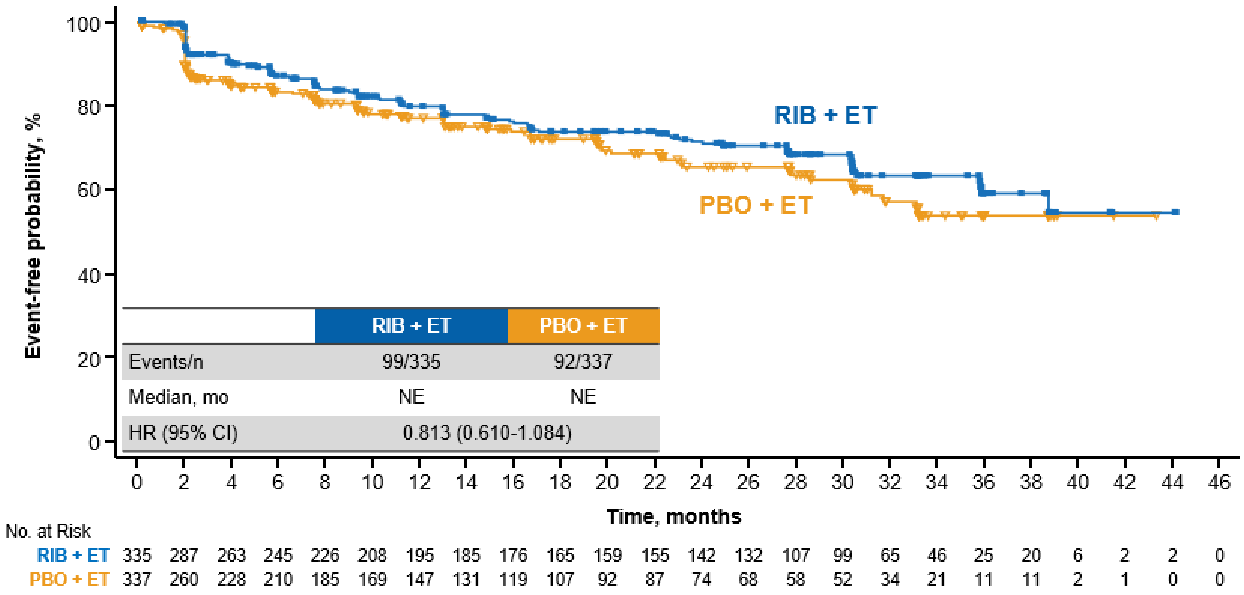

Supplemental Tables

Supplemental Table 1. Institutional review board or independent ethics committee listings

Center No.	Ethics Committee or Institutional Review Board[a]	Department / Organization	Address Country
1000	Eastern Health	Research Governance and Ethics	Box Hill VIC 3128 Australia
1001	Calvary health care Newcastle Limited	Research Governance and Ethics	Waratah NSW 2298 Australia

1002	St John of God health Care Human Research Ethics Committee	Human Research Ethics Committee	Wembley WA 6014 Australia
1003	Austin health	Human Research Ethics Committee	Heidelberg VIC 3084 Australia
1004	Bell berry Human Research Ethics Committee	Human Research Ethics Committee	Eastwood SA 5063 Australia
1200	Joint Chinese University of Hong Kong-New Territories East Cluster Clinical Research Ethics Committee	NA	Hong Kong NA NA Hong Kong
1201	Research Ethics Committee (Kowloon Central / Kowloon East)	NA	Hong Kong NA NA Hong Kong
1203	UNIMED Medical Institute Institutional Review Board	NA	Hong Kong NA NA Hong Kong
1301	Institutional Ethics Committee	NA	Bangalore Karnataka 560095 India
1302	Manavta Clinical Research Institute Ethics Committee	NA	Nashik Maharashtra 422001 India
1304	1. Institutional Ethics Committee 1, 2. Institutional Ethics Committee 2	NA	Mumbai Maharashtra India
1305	Ethical Committee	NA	Kolkata West Bengal 700053 India
1400	Institutional Review Board	Yonsei University Health System -Severance Hospital	Seoul NA 03722 Korea, republic of
1401	Seoul National University Bundang Hospital Institutional Review Board	Clinical Trial Center of Seoul National University Bundang Hospital	Seongnam-Si Gyeonggi-Do South Korea
1402	Institutional Review Board	Samsung Medical center	Seoul NA 06351 Korea, Republic of

1403	Asan Medical Center Institutional Review Board	Clinical Trial Center of Asan Medical Center	Seoul Seoul<br>South Korea
1404	Institutional Review Board	National Cancer center	Goyang-si Gyeonggi-do 10408<br>Korea, Republic of
1405	Seoul National University Hospital Institutional Review Board	Center for Human Research Protection of Seoul National University Hospital	Seoul Seoul 03080<br>South Korea
1500	Hammoud Hospital IRB	Hammoud Hospital	Saida Saida NA<br>Lebanon
1501	American University of Beirut IRB	American University of Beirut	Beirut Beirut NA<br>Lebanon
1502	Ethics Committee-Hotel Dieu De France	Saint Joseph University	Beirut Beirut NA<br>Lebanon
1503	Bellevue Medical Center IRB	Bellevue Medical Center	Beirut Beirut NA<br>Lebanon
1504	Saint Georges Hospital IRB	University of Balamand	Beirut Beirut NA<br>Lebanon
1505	Ain Wazein IRB	Ain Wazein Hospital	Al Chouf Al Chouf NA<br>Lebanon
1601	LEC under Arkhangelsk Clinical Oncology Dispensary	Arkhangelsk Oncology Regional Center<br>145/1, Obvodniy Kanal,<br>Arkhangelsk, Russia, 163045	Arkhangelsk NA<br>Russia
1603	LEC under BioEq	BioEq, LLC,<br>23, Krasnogvardeyskiy, per. Saint-Petersburg, Russia,<br>197342	Saint-Petersburg NA<br>Russia
1702	King Abdulaziz Medical City, KAIMRC, National Guard Health affairs	Biomedical Ethics section	Riyadh Central 22490<br>Saudi Arabia
1800	Centralised Institutional Review Board	CIRB B	NA NA<br>Singapore

1801	Centralised Institutional Review Board	CIRB B	NA NA Singapore
1900	Chulalongkorn Institutional Review Board	Faculty of medicine, Chulalongkorn University	Patumwan Bangkok Thailand
1901	Siriraj Institutional Review Board	Faculty of Medicine Siriraj Hospital, Mahidol University	Bangkoknoi Bangkok 10700 Thailand
2000	Research Ethics Committee, National Taiwan University Hospital	Research Ethics Committee C	Taipei NA 100 R.O.C
2001	Taipei Veterans General Hospital Institutional Review Board	Institutional Review Board	Taipei City NA R.O.C
2002	Taipei Medical University – Joint Institutional Review Board	NA	New Taipei City NA R.O.C
2003	Mackay Memorial Hospital Institutional Review Board	Institutional Review Board (2)	Taipei City NA R.O.C
2004	Institutional Review Board Committee<br>Koo Foundation Sun Yat-Sen Cancer Center	NA	Taipei City NA R.O.C
2005	Chang Gung Medical Foundation Institutional Review Board	Institutional Review Board A	Taipei City NA R.O.C
2006	Institutional Review Board Committee,<br>Changhua Christian Hospital, Taiwan	Institutional Review Board Committee A	Changhua NA 500 R.O.C
2007	Chang Gung Medical Foundation Institutional Review Board	Institutional Review Board A	Taipei City NA 10507 R.O.C

2100	İstanbul Üniversitesi Cerrahpaşa Tıp Fakültesi Klinik Araştırmalar Etik Kurulu	İstanbul Üniversitesi Cerrahpaşa Tıp Fakültesi Dekanlığı	İstanbul Cerrahpaşa 34098 Turkey
2101	İstanbul Üniversitesi Cerrahpaşa Tıp Fakültesi Klinik Araştırmalar Etik Kurulu	İstanbul Üniversitesi Cerrahpaşa Tıp Fakültesi Dekanlığı	İstanbul Cerrahpaşa 34098 Turkey
2102	İstanbul Üniversitesi Cerrahpaşa Tıp Fakültesi Klinik Araştırmalar Etik Kurulu	İstanbul Üniversitesi Cerrahpaşa Tıp Fakültesi Dekanlığı	İstanbul Cerrahpaşa 34098 Turkey
2103	İstanbul Üniversitesi Cerrahpaşa Tıp Fakültesi Klinik Araştırmalar Etik Kurulu	İstanbul Üniversitesi Cerrahpaşa Tıp Fakültesi Dekanlığı	İstanbul Cerrahpaşa 34098 Turkey
2104	İstanbul Üniversitesi Cerrahpaşa Tıp Fakültesi Klinik Araştırmalar Etik Kurulu	İstanbul Üniversitesi Cerrahpaşa Tıp Fakültesi Dekanlığı	İstanbul Cerrahpaşa 34098 Turkey
2105	İstanbul Üniversitesi Cerrahpaşa Tıp Fakültesi Klinik Araştırmalar Etik Kurulu	İstanbul Üniversitesi Cerrahpaşa Tıp Fakültesi Dekanlığı	İstanbul Cerrahpaşa 34098 Turkey
2200	Al Ain Medical District Human Research Ethics Committee	College of medicine UAE University	Al Ain Abu Dhabi NA United Arab Emirates
2500	Comite de Etica en Investigacion Clinica (CEIC)	NA	Buenos Aires Buenos Aires Larrea 1381 Dept 3 Floor Apt “A” – zip code: C1117ABK Argentina
2501	Comite Institucional de Etica de la Investigacion en Salud Clin. Reina Fabiola	NA	Cordoba Cordoba Oncativo 1248 – zip code: X5004FHP Argentina
2502	Comite de Etica en Investigacion Clinica (CEIC)	NA	Buenos Aires Buenos Aires Larrea 1381 Dept 3 Floor Apt “A” – zip code: C1117ABK Argentina
2600	Comite de Etica en Investigacion Clinica- Clinica del Country	Investigacion Clinica-Clinica del Country	Bogotá Cundinamarca 110221 Colombia

2601	Comite de Etica e investigaciones de Oncomedica S.A	Investigación Clínica-Oncomedica	Montería Cordoba 230017 Colombia
2700	Comité de Ética en Investigación de la Clínica Bajío CLINBA, S.C.	Comité de Ética en Investigación de la Clínica Bajío CLINBA, S.C.	Guanajuato Guanajuato 36090 Mexico
2706	Comité de Ética en Investigación de la Clínica Bajío CLINBA, S.C.	Comité de Ética en Investigación de la Clínica Bajío CLINBA, S.C.	Guanajuato Guanajuato Mexico
2709	Comité de ética en Investigación de la Escuela de Medicina del Instituto Tecnológico y de Estudios Superiores de Monterrey	Comité de ética en Investigación de la Escuela de Medicina del Instituto Tecnológico y de Estudios Superiores de Monterrey	Monterrey Nuevo León 64710 Mexico
2800	Comitê de Ética em Pesquisa da Fundação PIO XII- Hospital de Câncer de Barretos	NA	Barretos SP 14784-400 Brazil
2801	Hospital A.C. Camargo - Fundação Antonio Prudente Rua Professor Antonio Prudente, 211 – Liberdade	NA	Sao Paulo SP 01509-900 Brazil
2802	Comitê de Ética em Pesquisa da Universidade de Passo Fundo	Pró reitoria de pesquisa e pós graduação	Passo Fundo Rio Grande do Sul 99052-900 Brazil
2804	Comitê de Ética em Pesquisa da Universidade Regional do Noroeste do Estado do Rio Grande do Sul -Unijui	NA	Ijui Rio Grande do Sul 98700-000 Brazil

2805	Comitê de Ética em Pesquisa do Instituto Brasileiro de Controle do Câncer<br>Av. Alcântara Machado, 2576 – Mooca<br>Phone: (11) 3474-4227	Comitê de Ética em Pesquisa do Instituto Brasileiro de Controle do Câncer<br>Av. Alcântara Machado, 2576 – Mooca<br>Phone: (11) 3474-4227	São Paulo SP 03102-002<br>Brazil
2807	Comitê de Ética em Pesquisa do Centro de Referência da Saúde da Mulher-CRSM	Comitê de Ética em Pesquisa do Centro de Referência da Saúde da Mulher-CRSM	São Paulo SP 01317-010<br>Brazil
2809	Comitê de Ética em Pesquisa Envolvendo Seres Humanos-CEP/UEL<br>LABESC - Laboratório Escola - Rodovia Celso Garcia CId, Km 380 (PR445) - Campus Universitário	Comitê de Ética em Pesquisa Envolvendo Seres Humanos-CEP/UEL<br>LABESC - Laboratório Escola - Rodovia Celso Garcia CId, Km 380 (PR445) - Campus Universitário	Londrina PR 86057-970<br>Brazil
2900	UBC BCCA Research Ethics Board	Fairmont Medical Building	Vancouver BC V5Z1H8<br>Canada
2901	IRB Services 372 Hollandview Trail, Suite 300<br>Aurora,Ontario	NA	Aurora Ontario L4G 0A5<br>Canada
2902	Health Research Ethics Board of Alberta	Cancer Committee	Edmonton AB T5J 4A7<br>Canada
2903	OCREB (Ontario Cancer Research Ethics Board)	MaRS Center, 661 University Ave, Suite 510	Toronto Ontario M5G 0A3<br>Canada
2904	Comité d'éthique de la recherche<br>CHU de Québec - Université Laval	NA	Quebec QC G1L 3L5<br>Canada
2907	OCREB-Ontario Institute for Cancer Research<br>MaRS Centre 661 University Avenue, Suite 510	NA	Toronto Ontario M5G 0A3<br>Canada

3300	Medical Research Ethics Committee	University of Malaya Medical Centre	Kuala Lumpur Wilayah Persekutuan 59100<br>Malaysia
3301	Medical Research & Ethics Committee	Ministry of Health	Kuala Lumpur Wilayah Persekutuan 59000<br>Malaysia
4000	Commissie Medische Ethiek	UZ KU Leuven	Leuven NA 3000<br>Belgium
4001	Commissie voor Medische Ethiek	GZA Ziekenhuizen	Antwerpen NA 2018<br>Belgium
4002	Comité D' Ethique	Institut Bordet	Bruxelles NA 1000<br>Belgium
4004	Comité D' Ethique	Clinique et maternité Ste. Elisabeth	Namur NA 5000<br>Belgium
4100	Central IRB/IEC - Central Institutional Review Board/ Independent Ethics Committee	NA	Sofia Sofia<br>Bulgaria
4101	Central IRB/IEC - Central Institutional Review Board/ Independent Ethics Committee	NA	Sofia Sofia Bulgaria
4104	Central IRB/IEC - Central Institutional Review Board/ Independent Ethics Committee	NA	Sofia Sofia<br>Bulgaria
4170	CPP « Sud-Ouest et Outre-Mer III » - Bordeaux	Groupe Hospitalier Pellegrin<br>Service de Pharmacologie Clinique<br>Bâtiment 1A<br>Place Amélie-Raba-Léon	Bordeaux NA 33076<br>France
4171	CPP « Sud-Ouest et Outre-Mer III » - Bordeaux	Groupe Hospitalier Pellegrin<br>Service de Pharmacologie Clinique<br>Bâtiment 1A<br>Place Amélie-Raba-Léon	Bordeaux NA 33076<br>France

4172	CPP « Sud-Ouest et Outre-<br>Mer III » - Bordeaux	Groupe Hospitalier Pellegrin<br>Service de Pharmacologie Clinique Bâtiment 1A<br>Place Amélie-Raba-Léon	Bordeaux NA 33076<br>France
4173	CPP « Sud-Ouest et Outre-<br>Mer III » - Bordeaux	Groupe Hospitalier Pellegrin<br>Service de Pharmacologie<br>Clinique Bâtiment 1A<br>Place Amélie-Raba-Léon	Bordeaux NA 33076<br>France
4174	CPP « Sud-Ouest et Outre- Mer III » - Bordeaux	Groupe Hospitalier Pellegrin Service de Pharmacologie<br>Clinique Bâtiment 1A<br>Place Amélie-Raba-Léon	Bordeaux NA 33076 France
4176	CPP « Sud-Ouest et Outre-<br>Mer III » - Bordeaux	Groupe Hospitalier Pellegrin<br>Service de Pharmacologie Clinique Bâtiment 1A<br>Place Amélie-Raba-Léon	Bordeaux NA 33076<br>France
4177	CPP « Sud-Ouest et Outre-<br>Mer III » - Bordeaux	Groupe Hospitalier Pellegrin<br>Service de Pharmacologie Clinique Bâtiment 1A<br>Place Amélie-Raba-Léon	Bordeaux NA 33076<br>France
4178	CPP « Sud-Ouest et Outre-<br>Mer III » - Bordeaux	Groupe Hospitalier Pellegrin<br>Service de Pharmacologie Clinique Bâtiment 1A<br>Place Amélie-Raba-Léon	Bordeaux NA 33076<br>France
4200	Ethikkommission bei der<br>Medizinischen Fakultät der<br>LMU München	N/A	München Bayern 80336<br>Germany
4201	Ethikkommission an der<br>Medizinischen Fakultät der<br>Universität Leipzig	NA	Leipzig NA 04109<br>Germany
4203	Ethik-Kommission der<br>Medizinischen Fakultät der Christian-Albrechts-Universität zu Kiel	NA	Kiel NA 24105<br>Germany

4204	Ärztekammer Nordrhein Ethik-Kommission	NA	Düsseldorf NA 40474 Germany
4205	Ethikkommission der LÄK Baden- Württemberg	NA	Stuttgart NA 70597 Germany
4206	Ethikkommission an der Technischen Universität Dresden	NA	Dresden NA 01307 Germany
4207	Ethikkommission der Landesärztekammer Hessen	N/A	Frankfurt Hessen 60488 Germany
4211	Ethikkommission der Ärztekammer Nordrhein	NA	Düsseldorf NA 40474 Germany
4212	Ethikkommission der Universität Ulm	N/A	Ulm Baden-Württemberg 89081 Germany
4213	Ethikkommission der Medizinischen Fakultät der Friedrich-Alexander-Universität Erlangen-Nürnberg	N/A	Erlangen Bayern 91054 Germany
4215	Ethikkommission der LÄK Thüringen	NA	Jena NA 07751 Germany
4216	Landesärztekammer Baden-Württemberg Ethik-Kommission	NA	Stuttgart NA 70597 Germany
4300	Scientific Council of Euromedica General Clinic of Thessaloniki	Scientific Council	Thessaloniki Address: 11 Marias Kallas street 54645 Greece
4301	Scientific Council of the University General Hospital of Heraklion	Scientific Council	Heraklion Address: Voutes, Stavrakia 711 10 Greece
4400	Medical Research Council	Ethics committee for Clinical Pharmacology	Budapest NA 1051 Hungary
4401	Medical Research Council	Ethics committee for Clinical Pharmacology	Budapest NA 1051 Hungary

4402	Medical Research Council	Ethics committee for Clinical Pharmacology	Budapest NA 1051 Hungary
4403	Medical Research Council	Ethics committee for Clinical Pharmacology	Budapest NA 1051 Hungary
4405	Medical Research Council	Ethics committee for Clinical Pharmacology	Budapest NA 1051 Hungary
4406	Medical Research Council	Ethics committee for Clinical Pharmacology	Budapest NA 1051 Hungary
4500	Comitato etico centrale dell'irccs fondazione salvatore maugeri (irccs) di Pavia	Irccs fondazione salvatore maugeri (irccs) di pavia	Pavia pavia 27100 Italy
4501	Comitato etico indipendente dell'azienda ospedaliero-universitaria policlinico s.orsola-malpighi di bologna	NA	Via albertoni pietro, 15-padiglione 3 Bologna Bologna 40138 Italy
4503	Comitato etico catania 2 c/o azienda ospedaliera di rilievo nazionale e di alta specializzazione garibaldi (aornas garibaldi)	NA	Piazza santa maria gesù, 7 Catania Catania 95125 Italy
4505	Comitato Etico ASL Lecce	ASL- Lecce	Lecce Lecce 73100 Italy
4506	Comitato Etico dell'Istituto Nazionale Tumori Fondazione Pascale	Via Mariano Semmola	Napoli NA 80131 Italy
4507	Comitato Etico Lazio 1	Az Ospedaliera San Camillo Forlanini – Circonvallazione Gianicolense, 87	Roma RM 00152
4509	Comitato etico unico della provincia di ferrara c/o aou di ferrara	NA	Via aldo moro, 8 Ferrara Ferrara 44124 Italy

4511	Comitato etico area cremona, mantova e lodi c/o ao di cremona	NA	Via della concordia, 1<br>Cremona<br>Cremona 26100<br>Italy
4512	Comitato Etico Area Vasta Centro	Area Vasta Centro-Toscana	Firenze Firenze 50134<br>Italy
4513	Comitato etico regionale della liguria c/o irccs aou san martino-ist istituto nazionale per la ricerca sul cancro	NA	Largo rosanna benzi Genova<br>Genova 16132<br>Italy
4514	Comitato Etico IRCCS Candiolo SP142 Km 3.95<br>10060 Candiolo (TO)	NA	Candiolo - torino to 10060<br>Italy
4517	Comitato etico regionale (cer) delle marche c/o azienda ospedaliera universitaria ospedali riuniti umberto i-lancisi-gm salesi di ancona	NA	Via conca<br>Ancona<br>Ancona 60126<br>Italy
4518	CEAS Umbria	Umbria	Ellera di Corciano Perugia 06070<br>Italy
4519	Comitato etico irst irccs avr	NA	Via pieromaroncelli, 40<br>meldola<br>forli-cesena 47014<br>Italy
4522	Comitato etico per le province di l'aquila e teramo. presidio ospedaliero l'aquila, loc.vetoio, coppito	NA	L'aquila l'aquila 67100<br>Italy
4523	Comitato etico degli irccs istituto europeo di oncologia e centro cardiologico monzino	NA	Via giuseppe ripamonti, 435<br>Milano<br>Milano 20141<br>Italy

4524	Comitato etico regionale unico (ceru) c/o azienda ospedaliero-universitaria s.maria della misericordia di udine	NA	Piazzale s.maria della misericordia Udine<br>Udine 33100<br>Italy
4525	Comitato Etico AreaVasta Nord Ovest	AreaVasta Nord Ovest-Toscana	Pisa Pisa 56126<br>Italy
4526	Comitato Etico università Federico II di Napoli	Via Sergio Pansini, 5	Napoli NA 80131<br>Italy
4527	Comitato Etico fondazione Policlinico Universitario Gemelli	Policlinico Gemelli- Roma	Roma Roma 00168<br>Italy
4528	Comitato etico delle azienda sanitarie dell'umbria-segreteria scientifico-amministrativa del ceas umbria	NA	Via della rivoluzione, 16 – ellera di Corciano<br>Corciano<br>Perugia 06073<br>Italy
4601	Niezależna Komisja Bioetyczna ds. Badań Naukowych przy Gdańskim Uniwersytecie Medycznym	NA	Gdańsk NA 80-210<br>Poland
4602	Niezależna Komisja Bioetyczna ds. Badań Naukowych przy Gdańskim Uniwersytecie Medycznym	NA	Gdańsk NA 80-210<br>Poland
4700	Comissão de Ética para a Investigação Clínica (CEIC)	N/A	Lisbon Lisbon 1749-004<br>Portugal
4701	Comissão de Ética para a Investigação Clínica (CEIC)	N/A	Lisbon Lisbon 1749-004<br>Portugal
4702	Comissão de Ética para a Investigação Clínica (CEIC)	N/A	Lisbon Lisbon 1749-004<br>Portugal
4703	Comissão de Ética para a Investigação Clínica (CEIC)	N/A	Lisbon Lisbon 1749-004<br>Portugal

4704	Comissão de Ética para a Investigação Clínica (CEIC)	N/A	Lisbon Lisbon 1749-004 Portugal
4900	Commission Cantonale d'éthique et de la Recherche (CCER)	Hôpitaux Universitaires de Genève	Genève GE 1207 Switzerland
5000	Washington University Human Research Protection Office	NA	Saint Louis MO 63110 United States
5002	Office of the Human Research Protection Program (OHRPP)	NA	Los Angeles CA 90095 US
5004	MD Anderson Cancer Center Institutional Review Board	MD Anderson Cancer Center IRB	Houston Houston 77030 United States
5005	Quorum	NA	Seattle WA 98101 United States
5006	Quorum Review IRB	NA	Seattle WA 98101 United States
5010	Western Institutional Review Board	NA	1019 39th Avenue SE Suite 120 | Puyallup WA 98374--2115 United States
5011	Western Institutional Review Board	NA	Puyallup WA 98374 USA
5013	The Center For Cancer and Blood Disorders IRB	NA	Fort Worth Texas USA
5015	Western Institutional Review Board	NA	1019 39th Avenue SE Suite 120 | Puyallup WA 98374--2115 United States
5022	Quorum Review IRB	NA	Seattle WA 98101 USA
5024	Quorum Review INC	NA	Seattle WA 98101 USA
5025	University of Miami Institutional review board	NA	Miami FL 33136 USA
5027	Dana-Farber Cancer Institute IRB	NA	Valencia CA 91355 United States

5028	IRBMED	NA	Bldg 520 Room 3214,2800 Plymouth Rd Ann Arbor MI 48109 USA
5030	Memorial Healthcare System Institutional Review Board	NA	Hollywood FL 33021 United States
5033	Western Institutional Review Board	NA	1019 39th Avenue SE Suite 120 Puyallup WA 98374-2215 USA
5034	BSD IRB, The University of Chicago Biomedical Sciences Division/University of Chicago Medical Centre	5751 S. Woodlawn Ave, McGiffert House, 2nd Floor	Chicago IL 60637 USA
5040	Quorum IRB	NA	Seattle WA 98101 USA
5043	Kaiser Permanente Hawaii Institutional Review Board	NA	711 Kapiolani Blvd., Suite 110, Honolulu HI 96813 USA
5044	BAMC Institutional Review Board	NA	3551 Roger Brook Drive, Ft Sam Houston, TX 78234 TX 78234 USA
5045	Quorum Review IRB	NA	Seattle WA 98101 USA
5050	Western Institutional Review Board (WIRB)	1019 39th Avenue SE, Suite 120	Puyallup WA 98374-2115 USA
5061	Johns Hopkins Medicine Institutional Review Board	NA	Baltimore MD 21205-1911 USA
5062	Quorum Review IRB	NA	Seattle WA 98101 USA
5065	Houston Methodist Research Institutional Review Board	NA	Houston TX 77030 USA
5066	Penn State Milton S. Hershey Medical Center, Penn State College of Medicine	Human Subject Pretection Office	Hershey PA 17033 USA
5067	Western Institutional Review Board	NA	Puyallup WA 98374 USA

5068	Western Institutional Review Board	NA	1019 39th Ave SE, Puyallup WA 98374 USA
5073	Duke University Health System IRB	Duke University Medical Center, St. 405, Box 2712	Durham NC 27705 USA
5076	San Antonio Institutional Review Board	The University of Texas Health Science Center	San Antonio TX 78229-3900 USA
5078	Western Institutional Review board	NA	Puyallup WA 98374-2115 USA
5084	SCL Health-Front Range IRB	NA	1375 E. 19th Ave, Denver CO 80218 USA
5095	Western Institutional Review Board	NA	Puyallup WA 98374 USA
5096	WIRB	1019 39th Ave SE Suite 120	Puyallup WA 98374 USA
6000	CEIC Hospital Clínico San Carlos Doctor Martín Lagos, s/n. Puerta G - 4ª Norte	Hospital Clínico San Carlos Doctor Martín Lagos, s/n. Puerta G - 4ª Norte	Madrid Madrid Spain
6001	CEIC Hospital Clínico San Carlos Doctor Martín Lagos, s/n. Puerta G - 4ª Norte	Hospital Clínico San Carlos Doctor Martín Lagos, s/n. Puerta G - 4ª Norte	Madrid Madrid Spain
6002	CEIC Hospital Clínico San Carlos Doctor Martín Lagos, s/n. Puerta G - 4ª Norte	Hospital Clínico San Carlos Doctor Martín Lagos, s/n. Puerta G - 4ª Norte	Madrid Madrid Spain
6003	CEIC Hospital Clínico San Carlos Doctor Martín Lagos, s/n. Puerta G - 4ª Norte	Hospital Clínico San Carlos Doctor Martín Lagos, s/n. Puerta G - 4ª Norte	Madrid Madrid Spain

6004	CEIC Hospital Clínico San Carlos<br>Doctor Martín Lagos, s/n.<br>Puerta G - 4ª Norte	Hospital Clínico San Carlos<br>Doctor Martín Lagos, s/n.<br>Puerta G - 4ª Norte	Madrid Madrid<br>Spain
6005	CEIC Hospital Clínico San Carlos<br>Doctor Martín Lagos, s/n.<br>Puerta G - 4ª Norte	Hospital Clínico San Carlos<br>Doctor Martín Lagos, s/n.<br>Puerta G - 4ª Norte	Madrid Madrid<br>Spain

6006	CEIC Hospital Clínico San Carlos<br>Doctor Martín Lagos, s/n.<br>Puerta G - 4ª Norte	Hospital Clínico San Carlos<br>Doctor Martín Lagos, s/n.<br>Puerta G - 4ª Norte	Madrid Madrid<br>Spain
6007	CEIC Hospital Clínico San	Hospital Clínico San Carlos	Madrid Madrid
	Doctor Martín Lagos, s/n.<br>Puerta G - 4ª Norte	Puerta G - 4ª Norte	
6008	CEIC Hospital Clínico San Carlos<br>Doctor Martín Lagos, s/n.<br>Puerta G - 4ª Norte	Hospital Clínico San Carlos<br>Doctor Martín Lagos, s/n.<br>Puerta G - 4ª Norte	Madrid Madrid<br>Spain
6009	CEIC Hospital Clínico San Carlos<br>Doctor Martín Lagos, s/n.<br>Puerta G - 4ª Norte	Hospital Clínico San Carlos<br>Doctor Martín Lagos, s/n.<br>Puerta G - 4ª Norte	Madrid Madrid<br>Spain
6010	CEIC Hospital Clínico San Carlos<br>Doctor Martín Lagos, s/n.<br>Puerta G - 4ª Norte	Hospital Clínico San Carlos<br>Doctor Martín Lagos, s/n.<br>Puerta G - 4ª Norte	Madrid Madrid<br>Spain
6011	CEIC Hospital Clínico San Carlos<br>Doctor Martín Lagos, s/n.<br>Puerta G - 4ª Norte	Hospital Clínico San Carlos<br>Doctor Martín Lagos, s/n.<br>Puerta G - 4ª Norte	Madrid Madrid<br>Spain
6013	CEIC Hospital Clínico San Carlos<br>Doctor Martín Lagos, s/n.<br>Puerta G - 4ª Norte	Hospital Clínico San Carlos Doctor Martín Lagos, s/n.<br>Puerta G - 4ª Norte	Madrid Madrid Spain
6014	CEIC Hospital Clínico San Carlos<br>Doctor Martín Lagos, s/n.<br>Puerta G - 4ª Norte	Hospital Clínico San Carlos<br>Doctor Martín Lagos, s/n.<br>Puerta G - 4ª Norte	Madrid Madrid<br>Spain
6015	CEIC Hospital Clínico San	Hospital Clínico San Carlos	Madrid Madrid

	Carlos<br>Doctor Martín Lagos, s/n.<br>Puerta G - 4ª Norte	Doctor Martín Lagos, s/n.<br>Puerta G - 4ª Norte	Spain
6016	CEIC Hospital Clínico San Carlos<br>Doctor Martín Lagos, s/n.<br>Puerta G - 4ª Norte	Hospital Clínico San Carlos<br>Doctor Martín Lagos, s/n.<br>Puerta G - 4ª Norte	Madrid Madrid<br>Spain
6019	CEIC Hospital Clínico San Carlos<br>Doctor Martín Lagos, s/n.	Hospital Clínico San Carlos<br>Doctor Martín Lagos, s/n.<br>Puerta G - 4ª Norte	Madrid Madrid<br>Spain

[a] The study sponsor confirms that all of the IRBs/IECs listed approved the protocol and amendments.
